# Book Reviews

**Published:** 1908-07

**Authors:** 


### BOOK REVIEWS

---

SURGICAL DIAGNOSIS. By Daniel N. Eisendrath, M. D., Adjunct Professor of Surgery in the Medical Department of the University of Illinois (College of Physicians and Surgeons). Octavo of 775 pages, with 482 original illustrations, 15 in colors. Philadelphia and London: W. B. Saunders Company, 1907. Cloth, $6.50 net; Half Morocco, $8.00 net.

W. B. Saunders Company, Philadelphia and London.

Of first importance in every surgical condition is a correct diagnosis, for upon this depends the treatment to be pursued; and the two—diagnosis and treatment—constitute the most practical part of practical surgery. Dr. Eisendrath, in this superb new work, takes up each disease and injury amenable to surgical treatmen, and sets forth the means of correct diagnosis in a systematic and comprehensive way. The subject has been presented from a clinical standpoint, and the injuries and diseases grouped in the manner in which the surgeon or general practitioner considers them in examining the patient for the purpose of making a diagnosis. The importance of differentiating simulating affections has been constantly borne in mind, and every assistance given along these lines. Special effort, too, has been exerted to furnish the means of making a correct diagnosis in the early stages of the condition. Definite directions as to methods of examination are presented clearly and concisely, providing for all contingencies that might arise in any given case. The chapters on cystoscopy and urethral catheterization are unusually instructive. Dr. Eisendrath, being a strong advocate of the teaching of surgery by the education of the eyes has had specially made a large number of superb illustration. These four hundred and eighty-two pictures are not only artistic but practical, for each one gives practical assistance in diagnosing the condition under consideration. The work is beautifully gotten up.

---

PANAMA AND BACK. The record of an experience by Henry T. Byford, M. D., W. B. Conkey Company, Chicago.

This book is "dedicated to the Panama Canal Commissioners, who invited the President of the United States to run down and see them dig the canal while he waited; and to the President who went to the canal and found them asleep, and didn't wait until it was dug."

Dr. Byford has given an interesting description of his trip to Panama and the Fourth Pan-American Medical Congress of the Southern Surgical and Gynecological Association Banquet at Birmingham, the author gives a most complimentary description. He says it "constituted one of the most beautiful and intoxicating sights of the kind I have ever seen and partaken of, and led to the most exuberant five hou[illegible] of wit and humor of which I have personal knowledge * Hereafter I shall always speak of our Southern wit and humor as the most spontaneous and exuberant in the world. The North is witty because it is partly Irish, the South is wittier because it is entirely American."

ATLAS AND EPITOME OF DISEASES OF CHILDREN. By Dr. R. Hecker and Dr. J. Trumpp, of Munich. Edited, with additions, by Isaac A. Abt, M. D., Assistant Professor of the Diseases of Children in Rush Medical College, College, in affiliation with the University of Chicago. With 48 colored plates, 147 black and white illustrations, and 453 pages of text. Philadelphia and London: W. B. Saunders Company, 1907. Cloth, $5.00 net.

W. B. Saunders Company, Philadelphia and London.

Professors Hecker and Trumpp have provided an atlas of exceptional value. The many excellent lithographic plates represent cases seen in the authors' clinics, and have been selected with great care, keeping constantly in mind the practical needs of the general practitioner. The text, also, is admirable. Although concise, it covers the entire field of pediatric knowledge, and the editor, Dr. Isaac Abt, has added all new methods of treatemnt. Cloth, $5.00 net.

---

A MANUAL OF THE DIAGNOSIS AND TREATMENT OF THE DISEASES OF THE EYE. By Edward Jackson, M. D., Professor of Ophthalmology in the University of Colorado. Second Revised Edition. 12mo of 615 pages, with 182 text-illustrations and 2 colored plates. Philadelphia and London: W. B. Saunders Company, 1907. Cloth, $2.50 net.

W. B. Saunders Company, Philadelphia and London.

In this book more attention is given to the conditions that must be met and dealt with early in ophthalmic practice than to the rarer diseases and more difficult operations that may come later. It is designed to furnish efficient aid in the actual work of dealing with disease, and therefore gives the place of first importance to the conditions present in actual clinical work. A special chapter is devoted to the relations of occular symptoms and lesions to general disease. This second edition has been carefully revised.

INSOMNIA AND NERVE STRAIN. By H. S. Upson, M. D., Professor of Diseases of the Nervous System in the Western Reserve University. Attending Neurologist to the Lakeside Hospital, Cleveland, Ohio. With Skyagraphic Illustrations. G. P. Putnam's Son's. New York.

The object of the author of this book is to present a provisional sketch of the origin of the phychoses. The ultimate cause of many of the mental aberations are not all understood and Upson has made a special study of certain etiological factors, many of which, although apparently trivial in character, are of more importance than we have heretofore believed.

The view taken is that the neuroses and psychoses in general are primarily irritative disorders of the sensory system affecting the remainder of the nerve mechanism indirectly. Illustrative cases are given and mode of action and the location of the irritative lesions are taken up later. Especial stress is placed upon the subject of dental caries and imfaction of the teeth.

The subject is well presented and opens up an interesting field of thought.

